# Low Bone Mass in Probable CD1a-negative Langerhans Cell Histiocytosis: A Diagnostic Challenge

**DOI:** 10.1210/jcemcr/luaf211

**Published:** 2025-09-18

**Authors:** Rim Masri, Hussein Mokdad, Nermine Gherbewe, Batoul Raad, Zeinab Ali Issa

**Affiliations:** Department of Endocrinology, Faculty of Medical Sciences, Lebanese University, Hadath P.O. Box 3, Lebanon; Department of Internal Medicine, Faculty of Medical Sciences, Lebanese University, Hadath P.O. Box 3, Lebanon; Department of Internal Medicine, Faculty of Medical Sciences, Lebanese University, Hadath P.O. Box 3, Lebanon; Department of Neurosurgery, Faculty of Medical Sciences, Lebanese University, Hadath P.O. Box 3, Lebanon; Department of Endocrinology, Faculty of Medical Sciences, Lebanese University, Hadath P.O. Box 3, Lebanon; Department of Endocrinology, Al Rassoul Al Aazam Hospital, Beirut, Lebanon; Division of Endocrinology, American University of Beirut Medical Center, Beirut 1107 2020, Lebanon

**Keywords:** Langerhans cell histiocytosis, CD1a-negative, low bone mass for age, vertebra plana, young adult, bone lesions

## Abstract

Langerhans cell histiocytosis (LCH) is a rare disease characterized by CD1a/CD207-positive dendritic cells. This report presents a probable CD1a-negative LCH case in a 21-year-old man with chronic neck pain, low bone mass, and vertebra plana at the C6 level. Over 2 years, his symptoms progressed to severe vertebral collapse. Imaging and biopsy excluded malignancy and infection. Histopathology revealed a mixed immune infiltrate without CD1a expression, complicating diagnosis. LCH was confirmed through clinicoradiological correlation and exclusion of other disorders. Management included surgical stabilization and bisphosphonate therapy. This is the fourth reported CD1a-negative LCH case and the first associated with early-onset low bone mass for age. This case highlights diagnostic challenges and the importance of a multidisciplinary approach.

## Introduction

Langerhans cell histiocytosis (LCH) is a rare disorder of unknown etiology, characterized by clonal proliferation of CD1a- and/or CD207-positive dendritic cells [1, 2]. The term LCH, adopted in 1987, replaced *histiocytosis X*, originally coined by Lichtenstein in 1953 [2]. While LCH predominantly affects children, adult cases are increasingly reported. The annual incidence is 1.6 per million (4.46 in children, 1.06 in adults), with a higher rate in males [3].

LCH exhibits both neoplastic and inflammatory features, often forming granulomatous lesions in bone, skin, pituitary, lungs, spleen, and liver [1, 2]. In adults, 69% of cases involve multiple systems, most commonly the lungs and bones [4]. Bone involvement occurs in 80% of cases, particularly in the skull (37.5%), spine (26.5%), and long bones (14.1%), causing osteolytic lesions, fractures, and decreased bone mineral density (BMD) [1].

About 20% of adults with LCH have reduced BMD, increasing their risk of osteoporosis or osteopenia, especially in active disease or those older than age 50 years [5]. This study reports a rare case of CD1a-negative LCH in the cervical spine of a young adult male with low bone mass for age.

## Case Presentation

**Figure 1.**
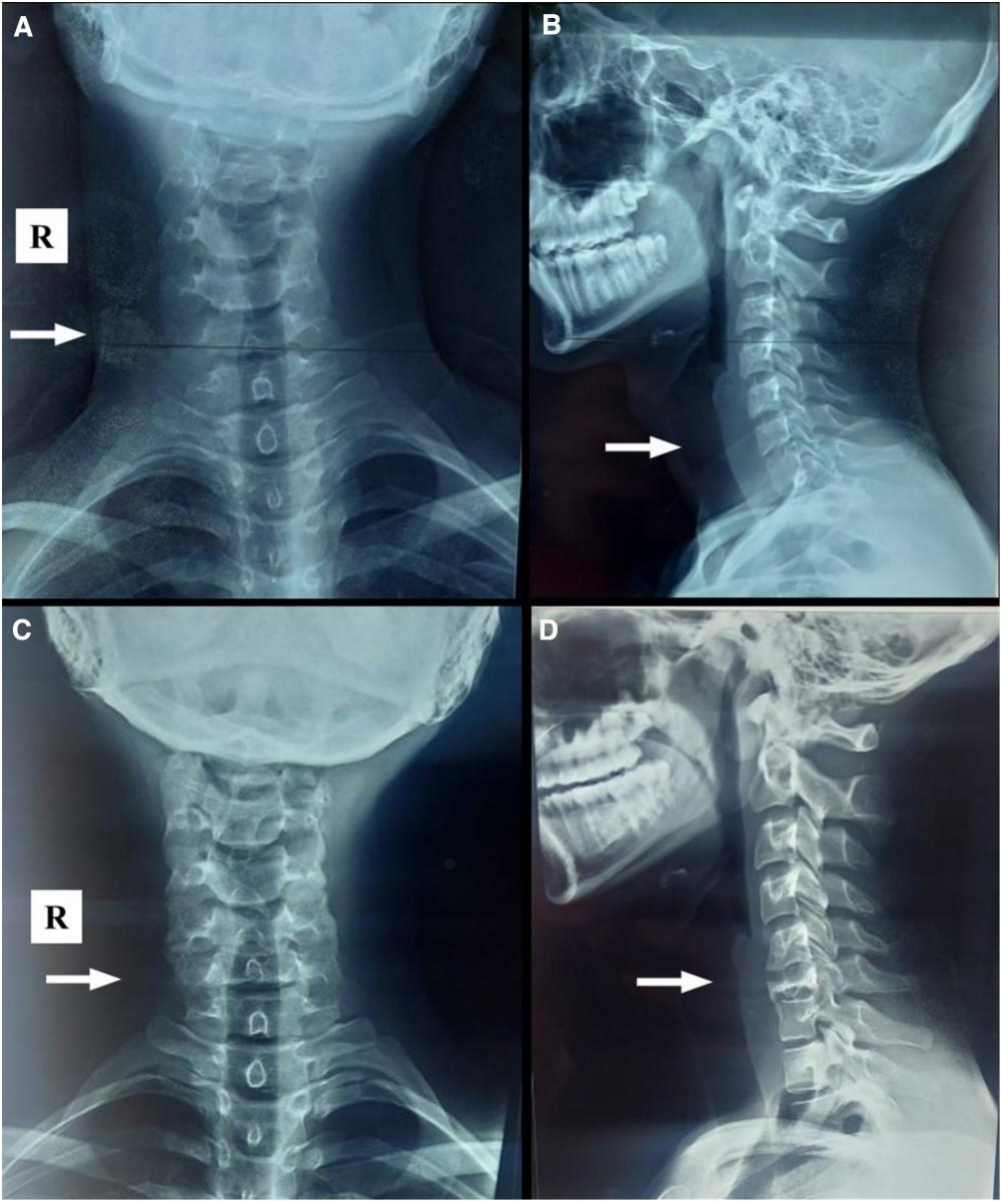
Cervical spine X-ray in anteroposterior and lateral views, demonstrating the progression from normal findings to a C6 vertebral body fracture. (A) Anteroposterior radiograph of the cervical spine obtained at initial presentation, showing normal alignment and vertebral morphology without evidence of fracture or degeneration. (B) Corresponding lateral view demonstrating intact vertebral bodies, preserved disc spaces, and normal cervical curvature. (C) Follow-up anteroposterior radiograph obtained 2 years later, revealing significant compression and degeneration of the C6 vertebral body. (D) Corresponding lateral view showing marked collapse and deformity of C6, consistent with vertebral compression fracture. White arrows indicate the C6 vertebral level in all images. R is for right side.

A previously healthy man presented to the primary care clinic at the age of 17 years with bothersome neck pain. Initially, he had no alarming symptoms and received nonsteroidal anti-inflammatory drugs and muscle relaxants. One month later, symptoms persisted, with no significant findings on the initial neck X-ray (Fig. 1A and 1B). Despite physiotherapy and chiropractic treatment, his pain intensified, affecting daily activities.

## Diagnostic Assessment

**Figure 2.**
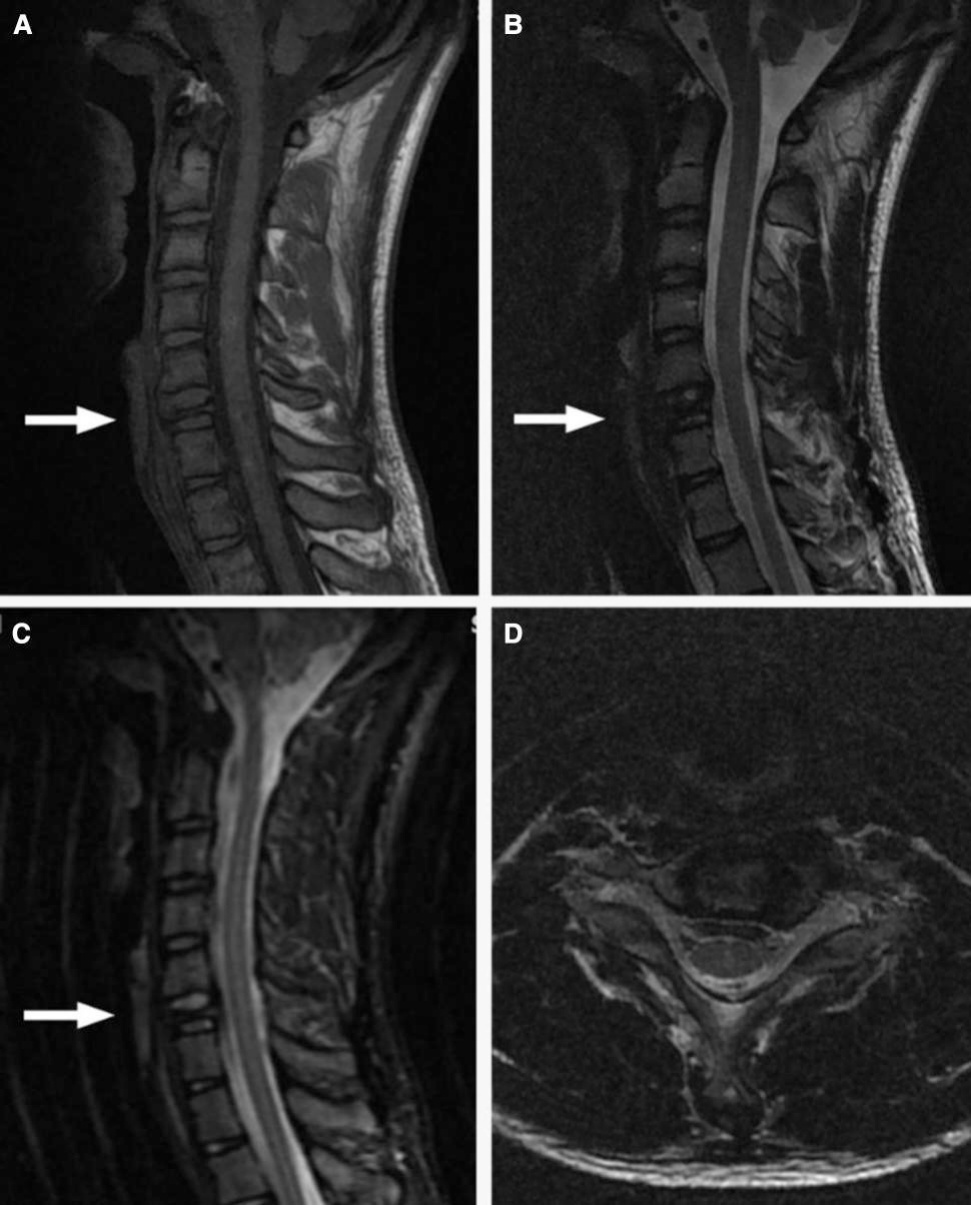
MRI of the cervical spine in multiple sequences and planes. Imaging demonstrates collapse of the C6 vertebra with approximately 90% loss of vertebral body height, consistent with vertebra plana, whereas the spinal cord remains unaffected. (A) Sagittal T1-weighted MR, (B) sagittal T2-weighted MRI, (C) sagittal short tau inversion recovery (STIR) MRI, and (D) axial T2-weighted MRI at the level of C6. In (A), (B), and (C), a white arrow indicates the level of the C6 vertebral body.

Two years later, he returned with persistent symptoms. A repeat X-ray revealed significant compression and degeneration of the C6 vertebral body (Fig. 1C and 1D). Magnetic resonance imaging (MRI) showed approximately 90% collapse of the C6, consistent with vertebra plana, with the spinal cord unaffected (Fig. 2). Thoracic and lumbar spine MRI scans showed no abnormalities.

Neurophysiological studies, including nerve conduction and needle electromyography, demonstrated chronic C7 radiculopathy without peripheral neuropathy. Positron emission tomography (PET) revealed a non-fluorodeoxyglucose-avid vertebra plana at C6 with no other active lesions, ruling out malignancy. Dual-energy X-ray absorptiometry (DXA) using a Hologic Horizon system recorded a lumbar spine *Z*-score of −2.6, consistent with low bone mass for age according to International Society for Clinical Densitometry criteria [6] (Table 1).

**Table 1.** **Dual-energy X-ray absorptiometry scan results**.

Region	BMD (g/cm^2^)	*Z*-score
Before treatment (HOLOGIC)		
1/3 Forearm	0.720 g/cm^2^	−1.3
Femoral neck	0.765 g/cm^2^	−1.3
Total femur	0.985 g/cm^2^	−0.3
L1-L4	0.769 g/cm^2^	**−2**.**6**
One year after bisphosphonate (GE Lunar)		
L1-L4	0.988 g/cm^2^	**−2**.**0**
Two years after bisphosphonate (GE Lunar)		
L1-L4	1.000 g/cm^2^	−0.7

Abnormal values are shown in bold font. Abbreviations: BMD, bone mineral density; GE Lunar, GE Lunar Prodigy Advance; HOLOGIC, Hologic Horizon DXA System; L1, lumbar vertebra 1; L4, lumbar vertebra 4.

The patient was evaluated for potential causes of pathological fractures. Laboratory tests including hormones, serum phosphate, calcium, vitamin D, and PTH were normal. Alkaline phosphatase was slightly elevated (Table 2). Serum protein electrophoresis was normal, and urine immunofixation showed no Bence Jones proteinuria. Infectious workup, including *Brucella* and tuberculosis serology, was negative.

**Table 2.** **Laboratory test results**.

Test	Result	Normal range
WBC	6.48 × 10³/μL (6.48 × 10⁹/L)	4.5-10.5 × 10³/μL (4.5-10.5 × 10⁹/L)
Neutrophils	57.5%	25-65%
Lymphocytes	32.6%	15-40%
Monocytes	7.6%	1-6%
Eosinophils	1.7%	0.6-4%
Basophils	0.6%	0-1%
RBC	**5.84** **×** **10⁶/μL (5.84** **×** **10¹²/L)**	4-5.5 × 10⁶/μL (4-5.5 × 10¹²/L)
Hemoglobin	17.7 g/dL (177 g/L)	12-18 g/dL (120-180 g/L)
Hematocrit	49.2%	37-52%
MCV	84.2 fL	78-97 fL
Platelets	318 × 10³/μL (318 × 10⁹/L)	150-450 × 10³/μL (150-450 × 10⁹/L)
ESR	2 mm/h	0-20 mm/h
Urea	21 mg/dL (3.5 mmol/L)	20-40 mg/dL (3.3-6.6 mmol/L)
Creatinine	0.9 mg/dL (79.6 µmol/L)	0.4-1.3 mg/dL (35.4-115 µmol/L)
Sodium	144 mEq/L (144 mmol/L)	135-145 mEq/L (135-145 mmol/L)
Potassium	4.7 mEq/L (4.7 mmol/L)	3.5-5.1 mEq/L (3.5-5.1 mmol/L)
Chloride	106 mEq/L (106 mmol/L)	95-112 mEq/L (95-112 mmol/L)
Calcium	10.3 mg/dL (2.57 mmol/L)	8.5-10.5 mg/dL (2.12-2.62 mmol/L)
Phosphate	3.6 mg/dL (1.16 mmol/L)	2.8-5 mg/dL (0.9-1.6 mmol/L)
Magnesium	2.03 mg/dL (0.835 mmol/L)	1.6-2.5 mg/dL (0.66-1.03 mmol/L)
C-reactive protein	0.4 mg/dL (4 mg/L)	0-1 mg/dL (0-10 mg/L)
*Brucella melitensis*	<1/40	Negative <1/40
*Brucella abortus*	<1/40	Negative <1/40
ALP	**92 U/L**	7-64 U/L
Ferritin	51.13 ng/mL (51.13 µg/L)	30-220 ng/mL (30-220 µg/L)
TSH	1.01 mIU/mL (1.01 mIU/L)	0.3-4.5 mIU/mL (0.3-4.5 mIU/L)
FT4	1.57 ng/mL (20.23 pmol/L)	0.93-1.7 ng/mL (12-22 pmol/L)
FSH	4.39 IU/L	1.5-11.8 IU/L
LH	11.8 IU/L	1.1-25 IU/L
Testosterone	497 ng/dL (17.2 nmol/L)	142.39-923.14 ng/dL (4.94-32 nmol/L)
PTH	48.8 pg/mL (48.8 ng/L)	15-69 pg/mL (15-69 ng/L)
25-OH vitamin D	44 ng/mL (110 nmol/L)	20-60 ng/mL (50-150 nmol/L)
Vitamin B12	602 pg/mL (444 pmol/L)	208-963.5 pg/mL (153-710 pmol/L)
Kappa FLC	12.32 mg/L	3.3-19.4 mg/L
Lambda FLC	12.14 mg/L	5.71-26.3 mg/L
Kappa/lambda ratio	1.01	0.26-1.65
Urine calcium (24 hours)	104 mg/24 h (2.6 mmol/24 h)	100-300 mg/24 h (2.5-7.5 mmol/24 h)
Urine creatinine (24 hours)	1461 mg/24 h (12.9 mmol/24 h)	600-2200 mg/24 h (5.3-19.5 mmol/24 h)

Abnormal values are shown in bold font. Values in parentheses are the International System of Units (SI).

Abbreviations: 24h, 24-hour urine collection; ALP, alkaline phosphatase; ESR, erythrocyte sedimentation rate; FLC, kappa free light chains; FT4, free thyroxine; MCV, mean corpuscular volume; RBC, red blood cells; WBC, white blood cells.

## Treatment

Based on these findings, bisphosphonate therapy (ibandronate) was initiated, along with calcium carbonate and calcifediol supplementation.

**Figure 3.**
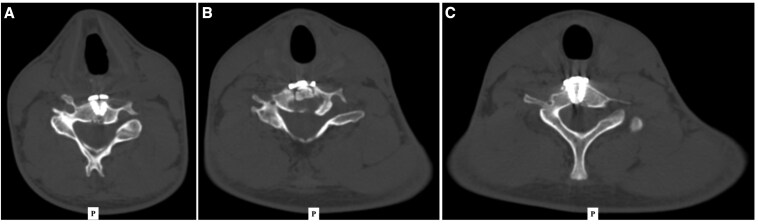
Postoperative axial CT scan images of the cervical spine at levels C5 (A), C6 (B), and C7 (C), following surgical fusion and stabilization. The patient underwent posterior cervical fusion of C5-C7 with pedicle screw fixation. All images demonstrate appropriate pedicule screw placement and preserved alignment. The letter “P” denotes the posterior direction for anatomical orientation.

Subsequently, the patient underwent cervical spine stabilization with C5-C7 fusion, including intraoperative biopsy of the C6 vertebral remnants (Fig. 3). The specimen consisted of fragments, the largest measuring 1.2 cm. Histopathology revealed bone lamellae separated by hypercellular marrow containing all three hematopoietic lineages, scattered medium-sized cells with mildly eosinophilic cytoplasm, and hyperplastic cartilage. Immunohistochemistry identified rare indeterminate cells and a reactive medullary space containing CD3+ T-lymphocytes, CD20+ B-lymphocytes, and CD138+ plasma cells. No expression was detected for CD1a, CD56, CD99, or Pan-Cytokeratin.

Considering the clinical presentation, age, radiologic findings, histopathology, and low bone mass, a diagnosis of probable CD1a-negative LCH, or LCH-like histiocytic disorder, was established. Because the lesion was localized and surgically resected, no specific systemic therapy for LCH was initiated. The patient was managed conservatively thereafter, with clinical and radiological monitoring.

## Outcome and Follow-up

Annual BMD assessments during bisphosphonate therapy showed progressive improvement in lumbar spine *Z*-scores, reaching −2.0 after 1 year and −0.7 after 2 years, measured using the same GE Lunar Prodigy Advance machine (Table 1).

## Discussion

This case describes a rare presentation of progressive cervical spine pathology in a previously healthy 21-year-old man, culminating in a pathological fracture and collapse of the C6 vertebral body. Symptoms began with nonspecific musculoskeletal pain treated conservatively; however, progressive worsening with red flag signs prompted further evaluation. Early MRI scans detected pathology before significant vertebral compromise, potentially improving prognosis [7]. The C6 collapse with preserved spinal cord function is highly unusual, suggesting a chronic rather than acute process. In young individuals, such a presentation warrants consideration of differential diagnoses including infectious causes (eg, spinal tuberculosis, brucellosis), primary or metastatic neoplasms (eg, lymphoma, Ewing sarcoma), metabolic bone diseases, and rare entities like LCH [8].

Unexplained progressive vertebral lesions in young patients require exclusion of rare serious pathologies [9]. This case posed a diagnostic challenge because of the broad spectrum of potential causes. Systematic workup investigated secondary osteoporosis and pathological fractures [10]. Brucella spondylitis was excluded by negative antibody titers for *Brucella abortus* and *melitensis*. Tuberculosis and malignancy were ruled out through histopathology and PET scan. In this case, the negative PET scan indicated metabolically inactive disease, supporting a nonaggressive or “burned-out” phase of LCH [11]. Hypogonadism, a known secondary cause of osteoporosis in males [12], was excluded by normal testosterone levels. Laboratory evaluation further ruled out other metabolic causes.

LCH is a rare but clinically important diagnosis in young patients with bone lesions or fractures [13], typically presenting with localized pain, edema, or pathological fractures [14]. MRI in this case revealed a 90% collapse of the C6 vertebra, whereas others were preserved. Typical spinal MRI findings in LCH include vertebra plana (complete collapse of the vertebral body), preserved intervertebral disc spaces, and absence of paraspinal soft tissue mass. In chronic or healing phases, lesions may show a sclerotic rim or bone remodeling, which helps differentiate LCH from malignancy or infection [1, 13]. Thoracic spine lesions are the most common in osseous LCH, followed by lumbar and cervical regions [1]; cervical involvement remains uncommon. Zhao et al reported that 26.5% of solitary bone LCH lesions involved the spine, with 88.2% affecting the vertebral body and exhibiting varying degrees of collapse [1]. Radiologic features such as vertebra plana, beveled-edge skull lesions, and isolated long bone diaphyseal destruction with fusiform periosteal reaction and surrounding edema, are strongly support LCH diagnosis [1, 13].

DXA in this patient confirmed low bone mass for age at the lumbar spine (*Z*-score −2.6), meeting International Society for Clinical Densitometry criteria (*Z* ≤ –2.0) for men younger than age 50 years and prompting evaluation for secondary causes. Baseline and follow-up scans were performed on different machines (Hologic vs GE Lunar), limiting direct comparability due to calibration and reference differences [6]. Despite apparent improvement in *Z*-score (−2.6 to −0.7), the modest 1.2% increase in absolute BMD suggests intermachine variability rather than true change. Careful review of original DXA images of our patient ruled out artifacts or fractures, underscoring the need for cautious interpretation across platforms [6]. Osteoporosis in LCH, particularly in young adults, is uncommon and may result from altered bone remodeling because of Langerhans cell infiltration, which stimulates osteoclast activity via inflammatory cytokines such as IL-1, TNF-α, and RANKL, leading to bone resorption [11]. Patients with active LCH have significantly lower lumbar spine and femoral neck *Z*-scores than matched controls and lower lumbar spine *Z*-scores than those with inactive disease, although osteoporosis-related fractures are rare [5]. In this case, the combination of low bone mass and vertebral collapse suggests a pathological process involving both LCH-related bone destruction and generalized bone loss.

A combination of clinical, laboratory, radiological, and histopathological criteria supported the diagnosis of probable LCH. Definitive diagnosis typically relies on immunohistochemical positivity for CD1a, S100, and Langerin (CD207) markers of Langerhans cells, as well as ultrastructural identification of Birbeck granules on electron microscopy [15, 16]. This case was challenging because of an absence of CD1a expression and unavailability of S100 and Langerin staining at our institution. Electron microscopy was not performed because of technical limitations and inadequate tissue preservation. Nevertheless, the diagnosis was supported by characteristic clinical and radiological features, including vertebra plana, and histologic findings of medium-sized cells with mildly eosinophilic cytoplasm in a reactive marrow background. These findings align with skeletal LCH and are supported by literature describing rare marker-negative presentations [13, 14, 17-20].

To date, only 3 cases of CD1a-negative LCH have been reported, highlighting the rarity of this variant: a 9-year-old boy with central nervous system involvement showing S100 positivity but CD1a negativity [18], a 35-year-old woman with skull lesions positive for S100 but negative for CD1a [19], and a 13-year-old girl with a frontal bone lesion negative for CD1a and S100 [20]. These cases vary in age, lesion site, and immunophenotypic profile, reflecting the diagnostic complexity of CD1a-negative LCH. Given the scarcity of reports, the true prevalence of this variant remains unclear. We believe our case represents the fourth reported instance and the first associated with early-onset low bone mass for age.

Alternative histiocytic disorders, including Rosai-Dorfman disease and Erdheim-Chester disease, which are typically CD68 positive [16, 21], were considered. Although CD68 staining was unavailable, the biopsy lacked emperipolesis, foamy histiocytes, and atypical infiltrates, and was negative for CD1a, CD56, CD99, and Pan-Cytokeratin. These findings make alternative diagnoses unlikely, though they cannot be entirely excluded without CD68 immunostaining. Although definitive diagnosis ideally requires positive immunohistochemistry and electron microscopy, LCH remains a clinicopathological diagnosis, particularly in rare immunonegative cases [15, 16, 21].

Managing osteoporosis in adult LCH is challenging because of its rarity and variable presentation. Treatment is largely extrapolated from pediatric protocols and tailored to disease severity and site involvement. In adults with multifocal bone or multisystem disease, chemotherapy remains a mainstay. Vinblastine, a vinca alkaloid, is widely used for its cytotoxic effects on proliferating Langerhans cells and is often combined with corticosteroids such as prednisone to enhance anti-inflammatory response and reduce lesion activity [22]. Corticosteroids alone may suffice in unifocal bone involvement, although combination therapy yields better outcomes in more aggressive forms [16]. Bisphosphonates, such as pamidronate or zoledronic acid, play a critical role in treating skeletal manifestations, particularly when osteolytic activity is prominent, as they inhibit bone resorption, reduce pain, and stabilize bone density in LCH-related bone loss [21].

In the absence of fractures, initiation of ibandronate therapy was justified by the markedly low bone mass (lumbar spine *Z*-score −2.6) in the context of LCH, a condition that compromises bone integrity and increases fracture risk [5, 11, 13]. Bone turnover markers (eg, CTX, P1NP, TRACP-5b) were not assessed because of institutional limitations, representing a gap in fully characterizing the patient’s bone metabolic status. Ibandronate was selected over other bisphosphonates based on local availability and patient preference for monthly intravenous administration, which was more practical than inpatient or more frequent infusion protocols.

The treatment aimed to prevent skeletal complications and preserve bone health in this high-risk setting. Its successful use in the present case supports evidence that bisphosphonates effectively decrease skeletal morbidity and improve BMD [23]. The overall approach in adults emphasizes symptom control, bone preservation, and prevention of complications such as fractures and deformities, especially in patients with osteoporosis.

Nutritional support is essential in managing skeletal complications in LCH, particularly in osteoporotic patients. Adequate vitamin D and calcium supplementation supports bone metabolism and helps counteract osteolytic activity [16]. Regular DXA monitoring is important to track bone density and guide treatment adjustments [11]. In the presented case, the patient remains under endocrinology follow-up, with routine DXA as part of ongoing care.

In conclusion, this case illustrates the diagnostic complexity of probable CD1a-negative LCH, a rare entity that may present with vertebral collapse and early-onset low bone mass in young adults. The absence of classical immunohistochemical markers necessitated a diagnosis through clinicoradiological and histopathological correlation, supported by a multidisciplinary evaluation. The cooccurrence of LCH and low bone mass highlights the importance of comprehensive assessment and long-term strategies to preserve bone health, including bisphosphonate therapy and nutritional support. Clinicians should maintain a high index of suspicion for atypical LCH in patients with unexplained skeletal lesions and bone loss, even when conventional histologic markers are absent.

## Learning Points

- CD1a-negative LCH is an exceptionally rare variant that may present with ambiguous histology.
- LCH should be considered in young adults with unexplained vertebral lesions and low bone mass for age.
- Diagnosis may require clinicoradiological correlation when immunohistochemistry is inconclusive.
- Low bone mass for age can be an early manifestation of skeletal LCH, even in young patients.
- Long-term follow-up, including bone health monitoring, is essential in LCH with skeletal involvement.

## Contributors

All authors contributed substantially to the development of this article. Z.I. supervised all tasks, provided direct clinical care, and oversaw patient management. R.M. designed the study, coordinated manuscript submission, and prepared tables and images. R.M., H.M., N.G., and B.R. collected data, performed the literature review, synthesized findings, and drafted the manuscript. All authors reviewed and approved the final version for submission.

## Data Availability

Original data generated and analyzed for this case report are included in this published article.
